# Experimental Study on Shear Characteristics of Filled Joints Anchored by Basalt Fiber-Reinforced Polymer Materials

**DOI:** 10.3390/ma18102393

**Published:** 2025-05-20

**Authors:** Hengjie Luan, Qingzhai Shi, Changsheng Wang, Yujing Jiang, Sunhao Zhang, Jianrong Liu, Kun Liu

**Affiliations:** 1 State Key Laboratory of Mining Disaster Prevention and Control Co-Founded by Shandong Province, Ministry of Science and Technology, Shandong University of Science and Technology, Qingdao 266590, China; luanjie0330@126.com (H.L.); 202282010062@sdust.edu.cn (Q.S.); jiangyjcn@gmail.com (Y.J.); lkun0311@163.com (K.L.); 2 Academician (Expert) Workstation, Inner Mongolia Shanghaimiao Mining Co., Ltd., Ordos 016299, China; 15048745086@163.com; 3 Graduate School of Engineering, Nagasaki University, Nagasaki 852-8521, Japan; 17854262633@163.com; 4 School of Civil and Environmental Engineering, Nanyang Technological University, Singapore 639798, Singapore

**Keywords:** mechanics experiment, rock joints, shear properties, filling degree, basalt fiber-reinforced polymer materials, acoustic emission, normal stress

## Abstract

Filled joints are widely found in natural rock masses and are one of the main factors causing rock mass engineering instability. The use of bolts can effectively control the shear slip of filled joints, research on bolts filled joints in the filling degree, and other key parameters of the influence of the law, to ensure the stability of the engineering rock body is of great significance. This paper presents shear experiments on bolted filled joints of Basalt Fiber-Reinforced Polymer (BFRP) materials with different joint roughness and filling degrees, while acoustic emission technology monitors the shear failure process of the specimens. The results show that the peak shear strength decreases with the increase in filling degree, and the peak shear strength decreases by 23.9% when the filling degree changes from 0 to 2.0 at 4 MPa and J2 conditions, while the normal stress, the Joint Roughness Coefficient (*JRC*) and the peak shear strength both show a positive correlation. The normal deformation of bolted filled joints exhibits three distinct evolutionary patterns depending on the filling degree, while both *JRC* and normal stress significantly influence the magnitude of shear dilatancy-shrinkage deformation. The shear resistance of BFRP bolts is mainly reflected in the post-peak plastic stage, and some of the fibers break during its shear deformation to form controlled yielding, with vertical and horizontal deformation controlled within 15.5~22.3 mm and 4.7~6.9 mm, respectively. The Acoustic Emission (AE) results show that the AE events are mainly in the post-peak plasticity stage, and the proportion is about the sum of the proportion of the other two phases, and this proportion increases with the increase in the filling degree.

## 1. Introduction

The existence of joint surfaces in the natural rock mass significantly reduces the mechanical properties of the rock mass, leading to the weakening of its deformation resistance and bearing capacity, which makes it more prone to instability destruction. A large number of engineering practices [1,2,3] show that, under the synergistic effect of ground stress and mining stress, the roadway surrounding rock is very easy to shear slip along the joint surface, which leads to the shear failure of the bolt and large deformation of the roadway surrounding rock and other phenomena. In recent years, with the increasing intensity and depth of coal resources mining, the instability of roadway surrounding rock induced by rock mass joints has become more and more prominent [4,5]. The bolt support is an effective means to control the roadway surrounding rock, and one of its main mechanisms is to limit the shear slip of the joint surface, which is fully affirmed by many support theories [6,7,8].

At present, the more commonly used type of bolt in roadway bolt support is steel material bolt, and many scholars at home and abroad have carried out a lot of research on its shear mechanical properties and bolt mechanism, etc. [9,10,11]. In terms of theoretical studies, researchers have investigated the bolting conditions [12], bolt types [13,14], *JRC* [12,15], bolting angles [16], and boundary conditions [17,18], respectively. In the field application of steel material bolts, Tao et al. [19] investigated the control of large deformation hazards of tunnel peripheral rock under different complex geological conditions by the active support method based on steel materials in engineering applications. Wang et al. [20] comprehensively verified the working performance of the synergistic bolting technique through field tests. The above exploratory studies on the shear properties and bolting mechanisms of steel material bolts in bolted joints have been fruitful. However, both these research results and practical engineering applications reveal a common phenomenon: steel material bolts are subjected to tensile-shear composite stresses and fracture is still frequent under small shear deformation [11,21]. Given this, systematic research on the key technology to improve the shear-breaking displacement of the bolt and in-depth discussion of its action mechanism is of great theoretical significance and engineering application value for improving the stability control effect of the joint rock mass [22,23].

In order to avoid the breakage of the bolt, which may cause a steep drop in shear stress and consequent instability of the surrounding rock, experts and scholars have carried out extensive research on new material bolts. Some researchers have optimized the traditional steel bolt materials; He et al. [24] carried out a study on the shear performance of joints bolted by NPR bolts, and it was found that NPR bolts can well balance the high strength and high ductility of conventional steel, and the tensile shear resistance is greatly improved. Wu et al. [25] investigated the shear characteristics of energy-absorbing bolts reinforcing rock mass under cyclic loading conditions by indoor shear experiments, and the results showed that the shear performance of the bolts in rock mass was strongly affected by cyclic shear loading, and the shear performance of energy-absorbing bolts was better than that of fully encapsulated bolts under cyclic shear loading conditions. Han et al. [26] conducted shear experiments on rock joints bolted by energy-absorbing bolts, and the results showed that energy-absorbing bolts have good ductility and can quickly respond to external load changes in the surrounding rock through their deformation or interaction with the surrounding rock to “absorb the energy” to inhibit the occurrence of bolt breakage.

In addition, basalt fiber-reinforced polymer (BFRP) bolt, as a new type of composite bolt, has the advantages of good tensile performance, corrosion resistance, fire resistance, and light weight, which is a better reinforcement material instead of steel reinforcement bolt for geotechnical engineering reinforcement [27,28,29]. Cao et al. [30] studied the mechanical properties of basalt fibers through experiments, and the results showed that the tensile strength of BFRP bar is more than twice that of ordinary steel bars, and the shear strength is only slightly less than that of steel bars, and the BFRP bar has better corrosion resistance. Zhang et al. [31,32] used an indoor direct shear experiment to compare and analyze the shear performance of BFRP bar and traditional steel bar bolted joint rock mass, BFRP bolt has low shear stiffness in elastic section and higher peak shear displacement, but higher residual shear strength compared with steel bar bolt. Xie et al. [33] carried out a double shear experiment on joint rock mass bolted by BFRP bolts to investigate the effect of bolting angle and preload on the shear capacity of bolted joints. Although a few scholars have carried out studies on the shear performance of bolted joints of BFRP bolts, the objects of their studies are all non-filled joints. In filled joints, the presence of soft fill significantly enhances the shear deformation and sliding tendency of the rock mass, which not only leads to a significant increase in the axial tensile force exerted on the bolts, but also greatly increases the likelihood of fracture of the bolts [34]. Unfortunately, there are still few reports on the research on the shear performance of BFRP bolts in bolted filled joints and their bolting mechanism, which seriously restricts the engineering application and promotion of BFRP bolts under complex geological conditions [35,36].

In view of the above understanding, the traditional steel material bolt breakage displacement is small and difficult to adapt to more complex surrounding rock conditions, while the BFRP bolts have the advantages of high tensile strength, corrosion resistance, and better coordination with the surrounding rock deformation ability. Therefore, this paper investigates the shear characteristics of bolted filled joints of BFRP bolts and the effects of normal stress, filling degree and joint roughness on their shear performance by carrying out the BFRP bolt bolted filled joint shear experiment. At the same time, acoustic emission technology is utilized to conduct dynamic nondestructive monitoring of the failure characteristics of bolted joint-filled specimens, thus elucidating the mechanism of interaction between BFRP materials and filled joints and breaking through the limitations of the bolting theory of the traditional steel material bolts. The research results not only provide theoretical basis for the bolting design of deep jointed rock projects but also provide technical support for the engineering application of new BFRP materials.

## 2. Shear Experiment

### 2.1. Specimen Preparation

**Figure 1:**
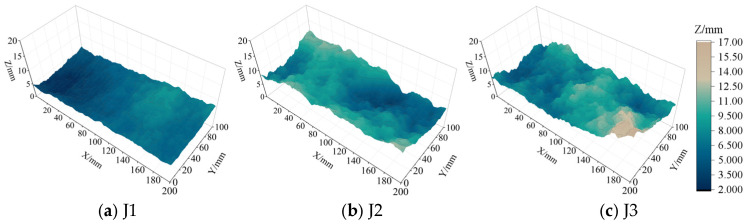
Surface topographies of rock joints with different *JRC*s.

In order to investigate the shear characteristics of BFRP bolted filled joints and the effects of normal stress, degree of filling and joint roughness on their shear performance, joint specimens with the same surface morphology are required. Therefore, this paper uses super-strong gypsum-like rock materials to prepare the specimens. First, the granite raw rock was cut into specimens with dimensions of 200 mm × 100 mm × 100 mm, and then it was split to obtain joint surface morphology, and the joint surfaces were reproduced on a silicone mold to cast rock-like specimens with the same joints. The nodal surfaces of the three different JRCs are shown in Figure 1. The joint roughness coefficients were calculated using the root mean square method of slope [37], where 50 profile lines were taken at equal intervals along the shear direction (x-direction) on the 3D rough joint surfaces, and the 2D *JRC* values of each curve were first calculated according to the following equation [15,26,38]:

(1) $$JRC=32.2+32.47\log\;Z_{2}$$

(2) $$Z_{2}={\left[\frac{1}{M}\sum {\left(\frac{z_{i-1}-z_{i}}{x_{i-1}-x_{i}}\right)}^{2}\right]}^{\frac{1}{2}}$$

where *Z*_2_ is the root mean square of the slope; *M* is the number of points on the curve; *x_i_* and *z_i_* are the coordinates of the points on the curve; the subscript *i* section is the number of times of selection, which is taken as 50.

Then, the average value of *JRC* of 50 curves is taken as the *JRC* value of the joint surface, and the calculation results yielded that the joint roughness coefficients of J1, J2, and J3 are *JRC*_1_ = 3.68, *JRC*_2_ = 6.18, and *JRC*_3_ = 9.86.

**Figure 2:**
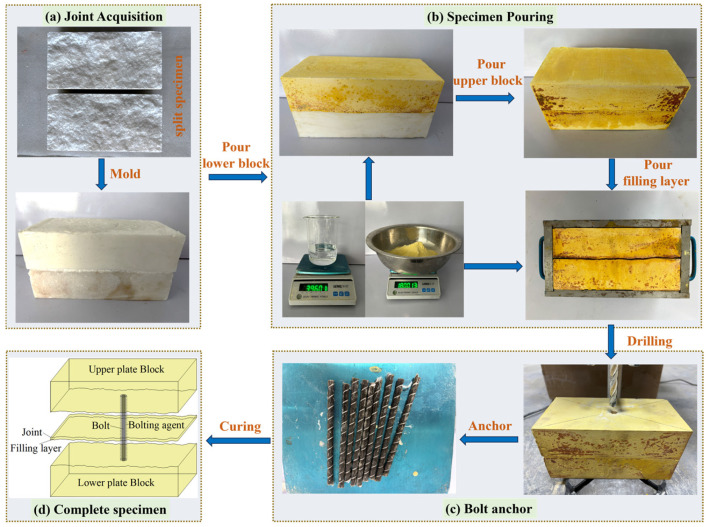
Preparation process of bolted filled joint specimens.

The complete specimen consists of three parts, which are the upper block, the lower block and the filling layer, and the specimen casting process is shown in Figure 2. Firstly, the upper and lower blocks were cast using silicone molds with replicated nodules surfaces; then, the cast upper and lower blocks were placed in the mold box to cast the bolted filled joints. Finally, after 2 weeks of maintenance of the cast specimens, circular bolt holes with a diameter of 10 mm were drilled and installed, and gypsum slurry was used as the bolting agent. After the specimens of bolted filled joints were made, they were further maintained for a fortnight and then experimented on.

Ladanyi et al. [39] proposed the concept of filling degree (Δ) to quantitatively describe the effect of filling thickness, which is numerically equal to the ratio of the filling thickness *t* to the difference in the surface relief of the rock joints *h*. The thickness of the filling layer during the casting of the specimen was obtained by calculating the degree of filling Δ and the difference in nodule surface undulation *h*.

**Figure 3:**
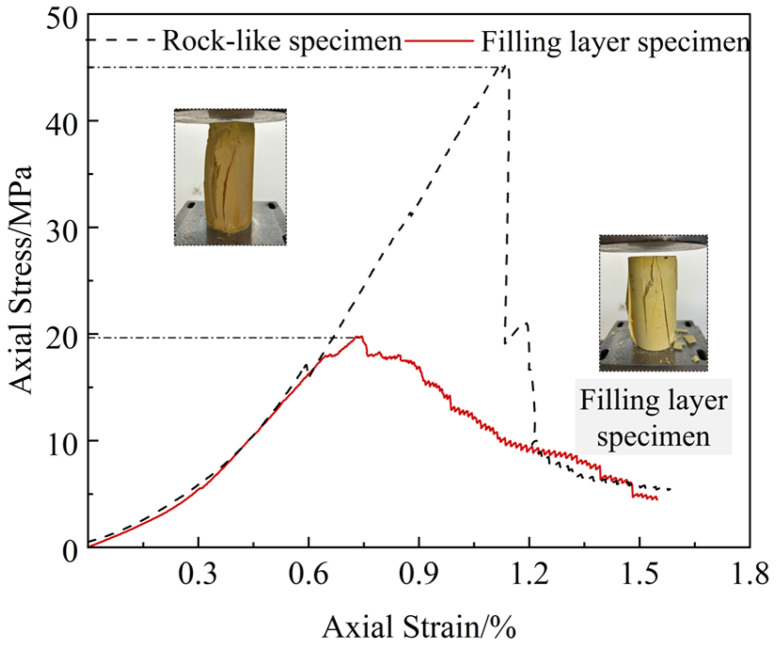
Mechanical property curves of similar materials in rock-like and filling layers.

The experiment ratio of the upper and lower blocks of the bolted filled joint specimen is high strength gypsum–water–retarded =1:0.22:0.0025, and the experiment ratio of the filled layer is high strength gypsum–water = 1:0.4. To experiment the basic mechanical parameters of the upper and lower blocks of rock materials and filling layer materials, cylindrical standard specimens were prepared according to the same ratios for uniaxial compression experiments, and their stress–strain curves and failure characteristics are shown in Figure 3. The uniaxial compressive strength and modulus of elasticity of the upper and lower blocks were 45.37 MPa and 5.11 GPa; the uniaxial compressive strength and modulus of elasticity of the filling layer specimens were 19.67 MPa and 2.96 GPa. The experiment bolts were made of 6 mm diameter basalt fiber composite bars with a tensile strength of 850 MPa, a modulus of elasticity of 58 GPa, an elongation of 2.6% and a density of 2.1 g/cm^3^, and ultimate shear strength of more than 150 MPa. BFRP bolts have excellent corrosion resistance to chloride ions, acid, and alkali and salt spray erosion; its coefficient of thermal expansion (6~8 × 10^−6^/°C) is close to that of concrete, which can effectively reduce structural damage caused by temperature stress.

### 2.2. Experimental Equipment

**Figure 4:**
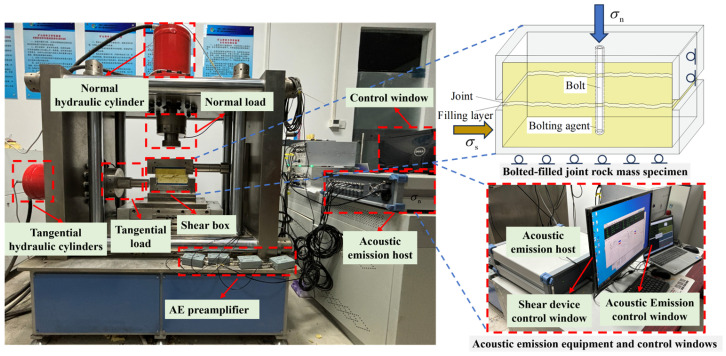
Shear experiment equipment.

The bolted filled joint shear experiment was carried out using the normal stiffness rock joint shear experiment system independently developed by Shandong University of Science and Technology, as shown in Figure 4. The experiment system consists of a shear mainframe, control system, oil source system, and manipulation system. The experiment system uses a hydraulic servo system to provide the working power, and the normal and tangential loading can reach a maximum of 600 kN. Real-time monitoring was performed using high-precision sensors with ±0.5% F.S. accuracy for both loading force and displacement measurements (normal and tangential directions), with data acquired at variable frequencies (5–50 Hz). During the experiment, the specimen was firstly placed in the shear box of the shear experimental system and the normal stress was applied to a constant value, and the normal stress was applied in a load-controlled mode with a rate of 0.1 kN/s; then the normal stress was kept constant, and the lower shear box was subjected to the shear stress, and the displacement-controlled mode was adopted, with a loading rate of 0.01 mm/s, and the test ended when the shear displacement was 10 mm.

**Figure 5:**
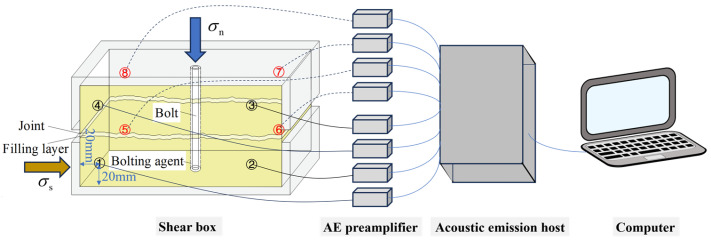
Acoustic emission connection schematic: The numbers 1–8 in the figure represent one AE sensor each, and the red font indicates the AE sensor on the reverse side of the specimen.

The DS5-16B full-information acoustic emission signal analyzer from Beijing Soft Island Times Technology Co Ltd. (Beijing, China) was used to monitor the acoustic emission signals of the bolt-filled joint specimens during the test process when they were subjected to shear damage, so as to realize the visual analysis of the cracks generated and expanded inside the specimens. Before the start of the experiment, four acoustic emission sensors were arranged on each of the two sides of the bolted filled joint specimen as shown in Figure 5, and the sensors were all arranged on the upper and lower blocks at a distance of 20 mm from the edge. The amplification of the acoustic emission front-end amplifier and the threshold of the system were set to 40 dB.

### 2.3. Experimental Scheme

The influence of filling degree is particularly significant among the factors affecting the shear mechanical properties of the rock mass of the filled joints [40]. Referring to the study by Song et al. [38], Δ = 0, 0.5, 1.0, 1.5, and 2.0 were determined, and the difference in the surface undulation of the J1, J2, and J3 joints, *h*, was derived from the surface morphology scanning data of the joints as 5 mm, 8 mm, and 11 mm. Generally, shear failure may occur when the normal stress ($\sigma_{n}$) is higher than 13% of the uniaxial compressive strength of the rock [41], so $\sigma_{n}$ was selected as 2 MPa, 4 MPa, and 6 MPa. The finalized scheme for the shear experiments of the bolted filled joint specimens is shown in Table 1.

## 3. Results of the Experiment

### 3.1. Shear Stress–Shear Displacement Curves

**Figure 6:**
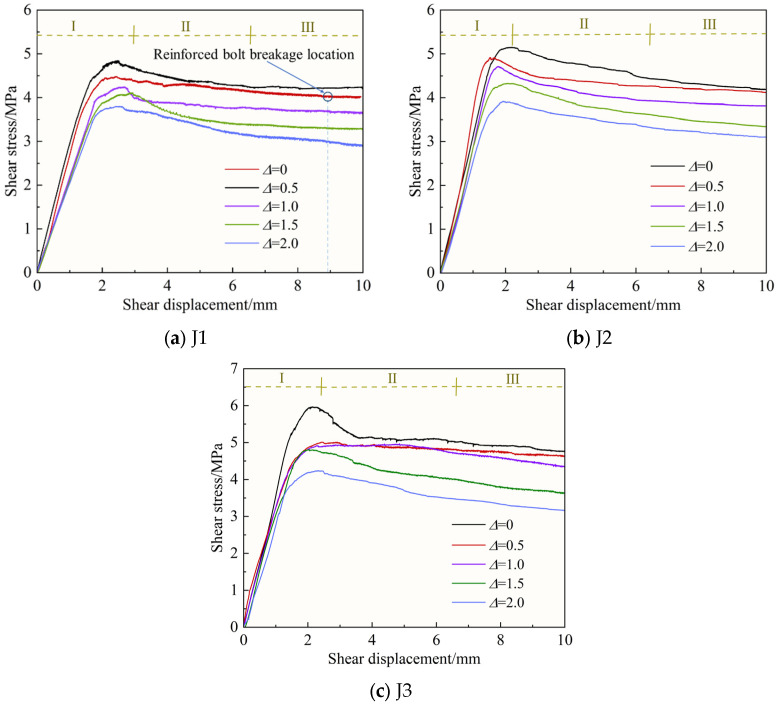
Shear stress–shear displacement curves: (I) Pre-peak elasticity Stage; (II) Post-peak plasticity Stage; (III) Residual strength Stage.

Limited to an extent, only the shear stress–shear displacement curves of the bolted filled joint specimens bolted by BFRP bolts under the condition of $\sigma_{n}$ = 4 MPa shown in Figure 6 are analyzed as an example. From the figure, it can be seen that the shear–stress displacement curve can be divided into three stages, which is essentially the same as the study of Zhang et al. [31], including the pre-peak elastic Stage (I), the post-peak plastic Stage (II), and the residual strength Stage (III).

Pre-peak elasticity Stage (I): This is the stage where the shear stress is approximately linearly related to the shear displacement, and the shear displacement is roughly in the range of 0~2 mm. In this stage, the specimen undergoes elastic deformation, the curve shape is approximately straight line, and the slope of the line is negatively correlated with the filling degree. The shear stress increases rapidly to the peak shear stress within a short range of shear displacement. As can be seen from Figure 6, the rougher the joint surface and the lower the degree of filling, the greater the peak shear stress, indicating that the magnitude of the peak stress is affected by the combination of multiple factors, such as the normal stress, the characteristics of the joint surface and the degree of filling.

Post-peak plasticity Stage (II): This is the stage at which the joint surface enters irreversible plastic deformation, and the shear displacement is roughly in the range of 2~6 mm. This phase starts with the shear stress rising to a peak value until the stress no longer changes significantly. Except for the specimen with Δ = 0, the peaks of the curves corresponding to the rest of the specimens are not obvious, which is due to the presence of the soft filling layer producing a significant buffering effect, making the bolted filled joint shear curves gentler. After the shear stress reaches the peak value, the bolted filled joint specimen has also reached the critical point of elastic and plastic deformation, and the curve enters the plastic deformation stage. With the increase in shear displacement, joint surface bulge and filling layer surface friction and extrusion failure, the bolt began to occur part of the basalt fiber breakage, to achieve the effect of “letting change”, but the whole still played the role of shear; bolted filled joint specimen bearing capacity decreased rapidly and, at the same time, the slope of the curve decreases, indicating that the shear stress tends to be gradually stabilized.

Residual strength Stage (III): This is the stage where the stress is stabilized at the residual strength, and the shear displacement is roughly in the range of 6~10 mm. At this stage, the shear stress no longer varies significantly with increasing shear displacement until the end of shear. In particular, the same gypsum slurry was used to prepare the bolted filled joint specimens for the shear experiment as in this paper, and the material ratios were similar, and Wu et al. [42] derived a breaking displacement of 8.891 mm for a reinforced bolt with *JRC* = 3.5 and a filling layer thickness of 3 mm, as shown in Figure 6a. In this paper, the similar J1, Δ = 0.5 BFRP bolt bolted filled joint specimens for the shear experiment, BFRP bolt at the end of the experiment dismantled found that only part of the basalt fiber breakage, still maintains better overall integrity, with a certain shear capacity.

### 3.2. Peak Shear Strength

Peak shear strength is an important parameter for analyzing the shear characteristics of bolted joints, which is affected by the combined effects of normal stress, joint surface roughness, rock and soft fill properties and other parameters. The specific values of the peak shear strength of the anchorage-filled joint specimens are shown in Table 2 and Table 3.

**Figure 7:**
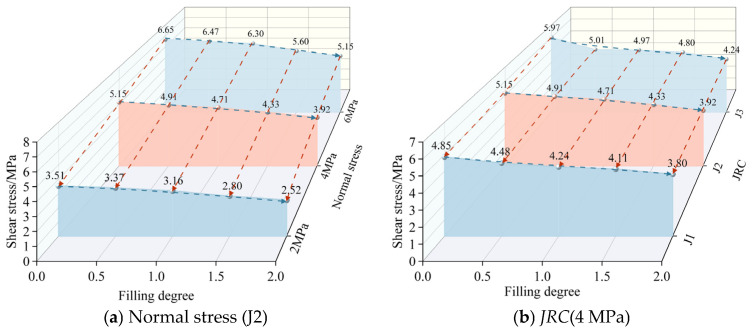
Characteristics of peak shear strength change.

In order to more intuitively reflect the variation in peak shear strength with normal stress and *JRC*, respectively, the data were summarized and presented in the form of 3D waterfall diagrams, as shown in Figure 7.

As can be seen in Figure 7, the peak shear strength exhibits a decreasing trend with increasing filling degree. Taking the J2 filled joint specimen in Figure 7b as an example, the peak shear strengths were 5.15 MPa, 4.91 MPa, 4.71 MPa, 4.33 MPa, and 3.92 MPa for the change in filling degree from 0 to 2.0. The strength of the filling layer is lower than that of the rock-like specimens, and with the increase in the filling degree, the contact between joint surfaces is gradually replaced by the filling material, resulting in a decrease in the overall shear strength; at the same time, the shear capacity of the filling layer is weaker, especially at high filling degrees, and the shear failure mainly occurs in the interior of the filling layer rather than the contact surface of the rock-like material and the filling layer.

Figure 7a shows the peak shear strength of the specimens of bolted filled joints under the condition of joint roughness J2. By comparing the peak shear strengths with the same filling degree, it can be seen that the increase in normal stress can significantly enhance the peak shear strength of bolted filled joints. $\sigma_{n}$ increased from 2 MPa to 4 MPa, the growth rates of peak shear strength were 46.7%, 45.7%, 49.1%, 54.6%, and 55.6% for the five filling degrees; $\sigma_{n}$ increased from 4 MPa to 6 MPa, the growth rates of peak shear strength were 29.1%, 31.8%, 33.8%, 29.3%, and 31.4% for the five filling degrees. It can also be seen that the promotion effect of normal stress on peak shear strength is more obvious at low normal stress when the growth is the same. This is due to the fact that the compaction effect of the filling layer is more significant at low normal stresses, and increasing the normal stress significantly improves the compactness and shear capacity of the filling layer; at high normal stresses, the filling layer is close to being fully compacted, and the effect of further increasing the normal stress is limited.

Figure 7b shows the peak shear strength of the bolted filled joint specimen at 4 MPa normal stress. As can be seen from the figure, the rougher the joint surface, the greater the peak shear strength of the bolted filled joint specimens, which is the same as the findings of Zhao et al. [40,41,43]. Taking Δ = 0 as an example, the peak shear strengths are 3.80 MPa, 3.92 MPa and 4.24 MPa for joint roughnesses J1, J2, and J3. However, due to the existence of the filling layer, the joint surface specimen changes from the original “rock-rock” surface contact to “rock-filling layer” surface contact, and the strength of the weak filling layer is obviously lower than that of the rock-like strength, and the “rock-filling layer” bumps are more prone to failure when subjected to the same force. The strength of the weak filling layer is obviously lower than the strength of the rock-like layer, and when the same force is applied, the “rock-filling layer” bulge is more likely to be broken, and the proportion of the resistance provided by a single bulge in the shear capacity of the joint surface decreases, which weakens the influence of joint roughness on the integrity of the specimen.

### 3.3. Normal Displacement–Shear Displacement Curves

**Figure 8:**
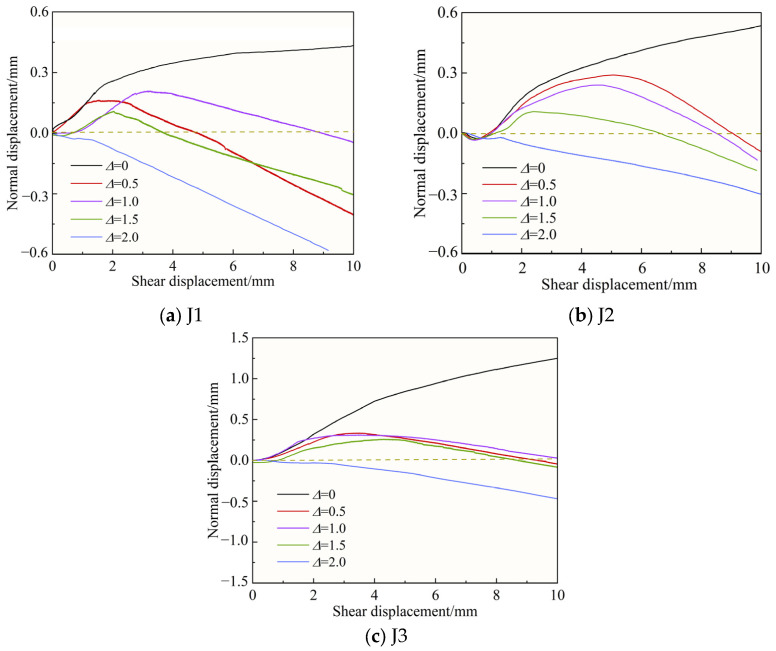
Normal displacement–shear displacement curves with different *JRC*s.

**Figure 9:**
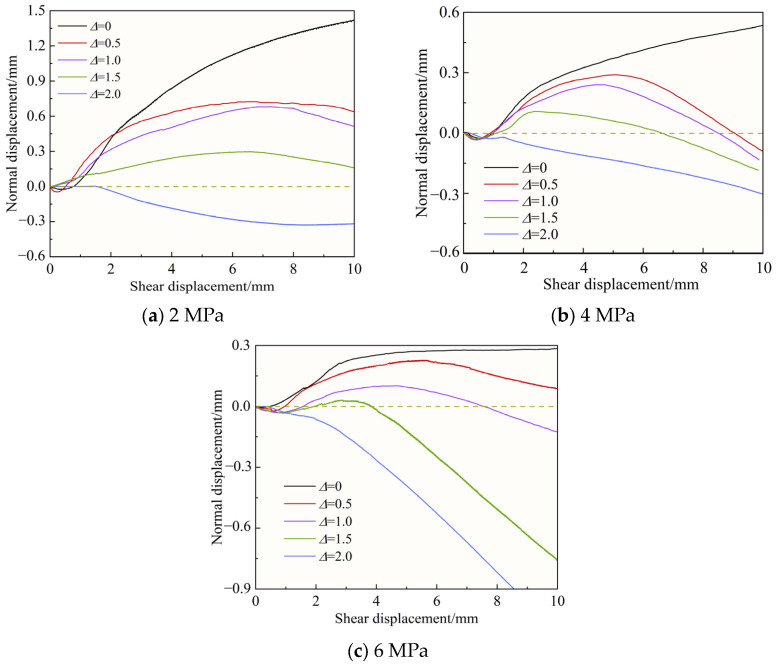
Normal displacement–shear displacement curves under different normal stresses.

**Figure 10:**
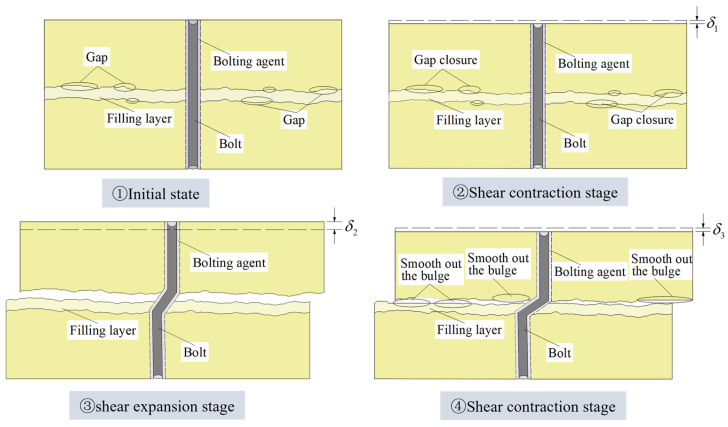
Schematic diagram of shear dilatancy-shear shrinkage for Δ = 1.0 bolted filled joint specimen.

Figure 8 shows the normal displacement–shear displacement relationship curves corresponding to different joint roughness coefficients (*JRC*) for normal stress $\sigma_{n}$ = 4 MPa. Figure 9 shows the normal displacement–shear displacement relationship curves corresponding to different normal stresses for J2 conditions. Figure 10 shows the shear dilatancy-shear contraction deformation of the specimen with filling degree Δ = 1.0 during the whole process of shearing under the conditions of normal stress $\sigma_{n}$ = 4 MPa and J2, where the dashed line represents the original height of the specimen and δ is the value of the normal displacement.

As can be seen from Figure 8, according to the different filling degrees, the change rule of normal displacement with shear displacement can be roughly classified into three kinds. When Δ = 0, the specimen first shear shrinkage deformation and then shear dilatancy deformation. The specimen is subjected to normal stress in the early stage of shear, and the gaps between joint surfaces are closed by compression, after which, with the increase in shear displacement, the climbing behavior of the joint surface bulge occurs, and although there is a shear failure of the joint bulge in the process, the whole is still manifested as shear dilatancy deformation. With the decrease in *JRC*, the degree of shear dilatancy gradually decreases. When Δ = 0.5, 1.0, and 1.5, the specimen is first shear shrinkage deformed, then shear dilatancy deformed and finally shear shrinkage deformed. The presence of the filling layer has an effect on the change in normal displacement compared to Δ = 0. As shown in Figure 10, after the filling joints were compacted after a short period of shear shrinkage deformation at the early stage of shear, the filling layer and the joint surfaces on both sides experienced climbing behavior along the bulge, which was manifested as shear dilatancy deformation; as the shear displacement continued to increase, the filling layer was crushed due to the bulge under the action of compression and shear with its low strength, and the specimen was subjected to shear shrinkage deformation. In particular, depending on the climbing behavior and the specific gravity of the bulge crushing, there are differences in the time for which the shear shrinkage and shear dilatancy deformations are maintained. When Δ = 2.0, the specimen has been kept in shear shrinkage deformation. Due to the greater thickness and lower strength of the filling layer, failure or fragmentation of the filling layer began to occur soon after the start of the shear experiment, the climbing behavior was not obvious, and the specimen underwent shear shrinkage deformation.

As shown in Figure 9, with the increase of $\sigma_{n}$, the degree of shear dilatancy deformation increases and shear shrinkage deformation slows down. Taking Δ = 0 as an example, the maximum normal displacements are 1.42 mm, 0.53 mm, and 0.28 mm at 2 MPa, 4 MPa, and 6 MPa. This is because the increase in normal stress aggravates the failure of joint bulge, the “climbing effect” becomes smooth, and the shear dilatancy is limited, at the same time, the increase in normal stress will lead to the more serious failure of filling layer, which provides space for the specimen to undergo shear deformation [44,45].

### 3.4. Characterization of Failure of Filled Joints

**Figure 11:**
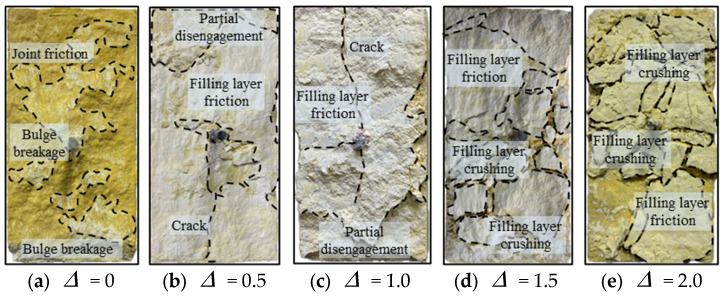
Figure of failure of specimens of bolted filled joints.

In order to better understand the bolted filled joint shear mechanical properties, the bolted filled joint failure characteristics were macroscopically analyzed [46]. Taking the joint surface J2 at $\sigma_{n}$ = 4 MPa as an example, its specimen failure is shown in Figure 11.

It is known from Figure 11 that when Δ = 0, the specimen is in “joint-joint” surface contact, and the joint surface undergoes friction and bulge gnawing by the shear action. When Δ = 0.5 and Δ = 1.0, due to the presence of weak filler, the specimen is “joint-filling layer” surface contact, but due to the small thickness of the filling layer, there will be a small area of “joint-joint” surface contact in the shear process. Therefore, the specimen mainly suffers from the friction of the filling medium and the whole detachment of part of the filling medium, accompanied by the friction and gnawing failure of fewer joint bulges. When Δ = 1.5 and Δ = 2.0, the thickness of the weak filling is larger, the contribution of the joints to the shear capacity is limited, the filling layer and the bolt mainly bear the shear effect of the specimen, under the joint action of the normal stress and the shear stress, the filling layer is more likely to be a failure than the upper and lower blocks and the fragmentation is more obvious.

### 3.5. Characterization of Failure of BFRP Bolt

**Figure 12:**
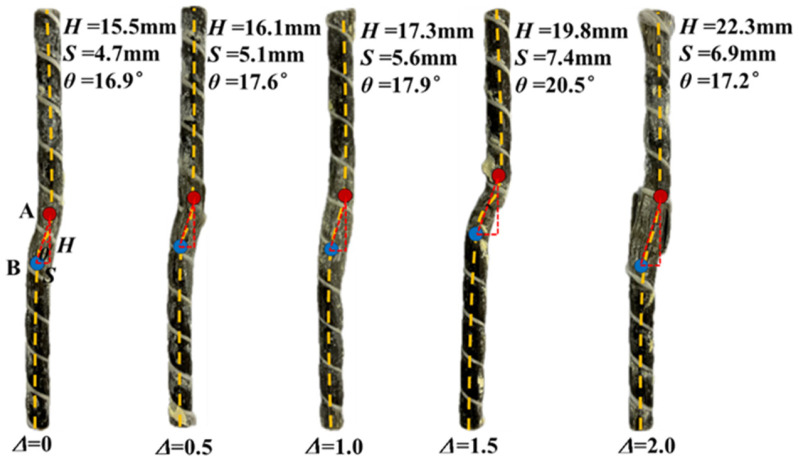
Deformation failure figure of BFRP bolt: A and B both indicate the turning point where the bolt undergoes deformation.

The failure characteristics of the BFRP bolt under normal stress of 4 MPa with different filling degrees are shown in Figure 12; A and B both indicate the turning point where the bolt undergoes deformation, *θ* denotes the bending angle of the bolt, and *H* and *S* denote the vertical and horizontal components of the bending and deformation part of the bolt. In this paper, we focus on analyzing the effect of the filling degree and the laws governing the effects of normal stress and roughness on bolted joint roughness have been revealed [47,48] and will not be repeated here.

**Figure 13:**
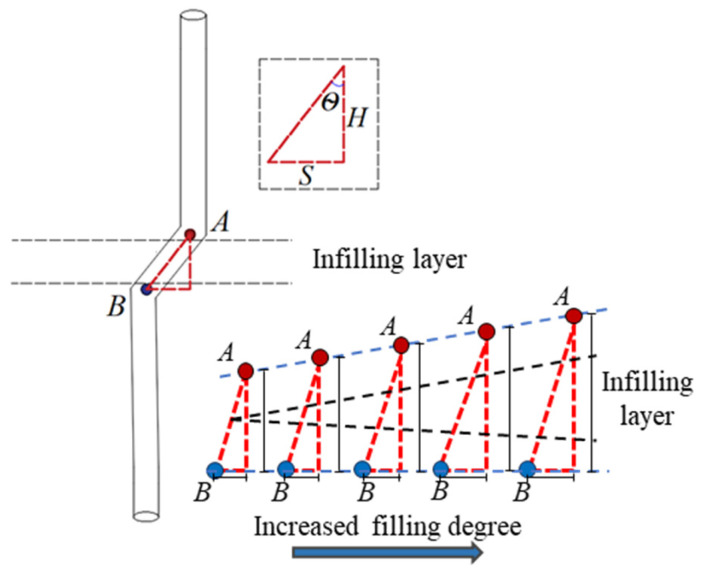
Schematic diagram of the geometric relationship between shear deformation of the bolt.

**Figure 14:**
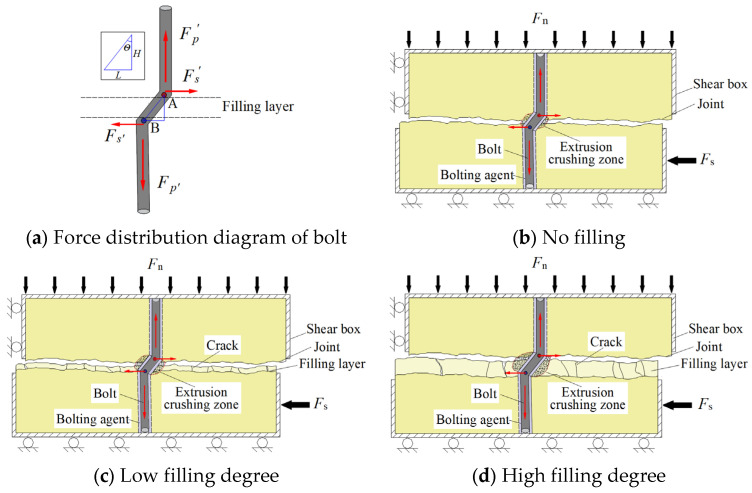
Schematic diagram of the failure of the bolt under force.

From Figure 12 and Figure 13, it can be seen that the BFRP bolts showed a slight “*Z*” bending deformation during the shear process, and with the increase in the filling degree, the deformation and failure area and bending degree of the bolts were enlarged. This is due to the low strength of the filling layer, the part of the layer in contact with the bolt is easily crushed by the extrusion pressure during the shear process and forms an extrusion crushing zone. Therefore, it is difficult for the filling layer to exert shear force on the bolt; it can be considered that the contact point between the upper and lower nodal surfaces and the bolt is the point of action of shear stress, as shown in Figure 14. The normal force on the BFRP bolt Fp' is the bonding constraint force exerted by the bolting agent, which is expressed as the bolt is subjected to the tension force exerted by the upper block specimen upward along the surface of the bolt and the tension force exerted by the lower block downward along the surface of the bolt. The bolt is subjected to the shear force Fs' in the horizontal direction in the region of the bolted filled joints, and $F_{n}$ denotes the force exerted on the shear box in the normal direction. Therefore, most of the turning points of the bolt deformation exist at the junction of the upper and lower blocks and the filling layer, and with the increase in the filling degree, the distance between points A and B increases, and the range of the bolt deformation expand.

BFRP bolts are virtually free from shear throughout the pre-peak elastic stage. After entering the post-peak plasticity stage, the shear force on the bolt increases rapidly, and the basalt fiber absorbs a lot of energy and is elongated, with the increase in the stretching length of the basalt fiber, a small portion of the fiber appears to be worn out, broken phenomenon, but overall, it continues to play a role; this achievement has the effect of “letting change”. In the residual strength stage, the shear displacement is larger, and the bolt is more exposed to the tensile load caused by the misalignment of the filled joints in the shear residual stage, while the BFRP bolt is more resistant to tensile ability and therefore shows a greater advantage in the residual stage. The BFRP bolt has a low modulus of elasticity and shows high elastic deformation capacity when subjected to force. During the shear process of filling joints, the BFRP bolt undergoes significant deformation when subjected to large tensile and shear forces, but it is not easy to fracture, which ensures that the BFRP bolt continues to play a shear-resistant role. However, it can be seen in Figure 12 that the horizontal deformation of the BFRP bolt, *S*, ranges between 4.7 mm and 6.9 mm, which is less than the set maximum shear displacement of 10 mm. This is due to a certain degree of deformation recovery of the BFRP bolt after the release of pressure and unconfinement of the rock-like material.

## 4. Acoustic Emission Characterization

### 4.1. Characteristics of AE Energy Changes

The AE energy is closely related to the specimen failure and can be used to characterize the specimen failure [49,50]. The shear stress–shear displacement curves and energy change curves under different filling degrees, normal stress and *JRC* conditions are listed, respectively, and the specimen failure law is described after comparative analysis.

In the pre-peak elastic phase, due to the elastic deformation of the specimen, the bolted filled joint specimen is basically undamaged, the AE signal is calmer, and the AE energy is smaller. In the post-peak plastic phase, the bolted filled joints reached the yield strength and began to undergo plastic failure, which was manifested by the friction failure of the filled joint surfaces, the fracture of the basalt fibers of the bolts, and the extrusion failure of the bolts against the filled joints, which resulted in a significant enhancement of the AE signals, and the AE energy increased rapidly to the peak. Afterwards, due to the gradual reduction in the number of projections and the gradual formation of the crush zone, the AE energy shows a general decreasing trend. In the residual strength stage, the interface of bolted filled joints is smoother, the deformation of the BFRP bolt is basically stable, the plastic failure is relatively less, the AE energy is lower, and the cumulative AE energy curve is smoother.

**Figure 15:**
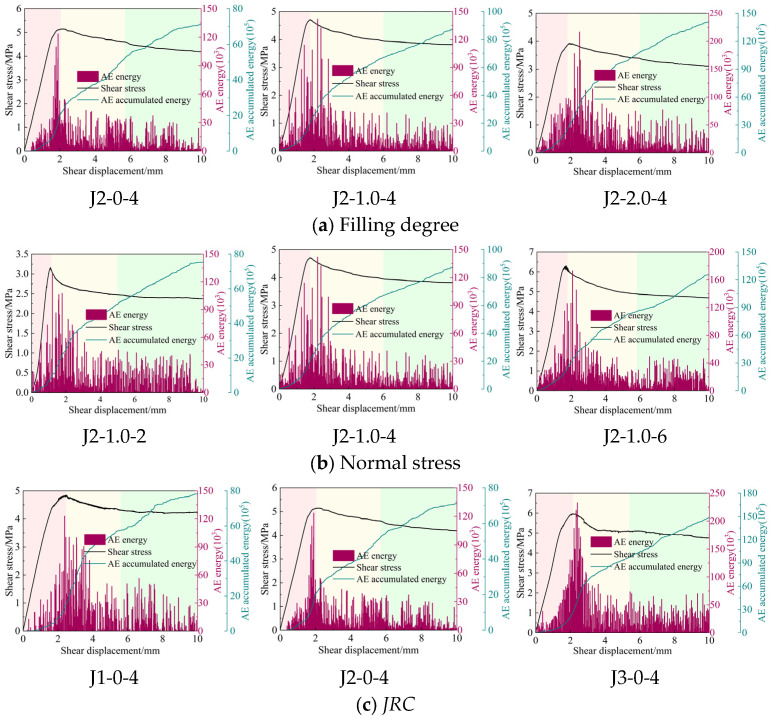
Characteristics of AE energy changes in bolted filled joint specimens.

The AE energies show different patterns as influenced by the soft fill. When Δ = 0, i.e., the specimen has no weak filling layer, the failure is dominated by brittleness with less energy release, while Δ ≠ 0, i.e., when there is a filling layer, due to the lower strength of the filling layer, it is prone to plastic deformation and rupture, and the failure mode of the filled joint specimen is gradually transformed into plastic failure with more significant energy release. At the same time, with the increase in filling degree, the volume of the filling layer increases, and the range and intensity of energy release in the shear process also increases. From Figure 15a, the maximum values of AE energy are 122, 142, and 216 (×10^3^ mV·ms), and the cumulative values of AE energy are 70, 87, and 141 (×10^5^ mV·ms) when Δ = 0, 1.0, 2.0.

The normal stress also has an effect on the AE energy values, with both the AE energy and the cumulative energy increasing as the normal stress becomes larger. From Figure 15b, the maximum values of AE energy are 128, 142, and 173 (×10^3^ mV·ms), and the cumulative AE energy values are 75, 87, and 125 (×10^5^ mV·ms) for normal stresses equal to 2, 4, and 6 MPa. This is due to the fact that the normal stresses can enhance the degree of engagement of the filled joints, the more severe failure occurs in the specimen under shear, and the AE energy released increases. In addition, the increase in *JRC* leads to more severe joint surface bulge failure and the release of instantaneous AE energy from bulge fracture increases.

### 4.2. AE Event Distribution Characteristics

The spatiotemporal distribution characteristics of AE signals in bolted filled joint specimens reflect the failure morphology of the specimens. Statistics on the distribution of AE signals and analysis of the connection between the distribution characteristics of AE signals and the failure of the specimens are conducive to analyzing the failure mechanism of the bolts and improving the bolting performance of the bolting system.

**Figure 16:**
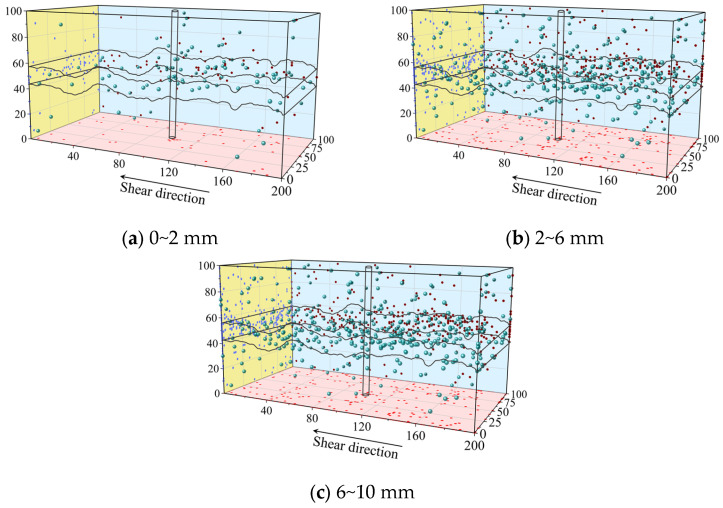
Characteristic figure of the spatial distribution of AE in the specimens of bolted filled joints: The green ball represents the AE signal; the red, blue and purple balls represent the projections of the AE signal in the three directions.

Figure 16 lists the characteristic figures of AE spatial distribution for different shear stages of the J2-2-4 bolted filled joint specimen, where each AE signal represents a failure event. From the figure, it can be seen that most of the AE events at small shear displacements tend to occur at the back end along the shear direction, and the AE events generated at this stage are caused by the closure of primary fractures and microcrack extension within the rock mass, which is the same as the study of Dong [51]. With the increase in shear displacement, the filling joint crack sprouting and expansion gradually increased and moved forward, and the AE events were uniformly distributed along the shear direction on the filling joint surface, and, exceptionally, the AE signals around the bolts were more intensive, which was caused by the formation of fracture zones in the rock mass around the extrusion of the bolts, as shown in Figure 16b. The AE signals increased after that, but the number was not large. It can also be seen from the figure that AE events occurred mostly at shear displacements of 2~6 mm (200~600 s), while there were fewer AE events at 0~2 mm (0~200 s) and 6~10 mm (600~1000 s).

As can be seen from the side projection, the AE signals are concentrated in the filling layer and near the joint surface, and there are fewer AE signals in the upper and lower blocks. In connection with the previous analysis, the filling joints are more prone to failure as a weak structure of the intact specimen as compared with the upper and lower rock specimens, and there is also a small amount of microcracks sprouting and expanding inside the upper and lower blocks.

**Figure 17:**
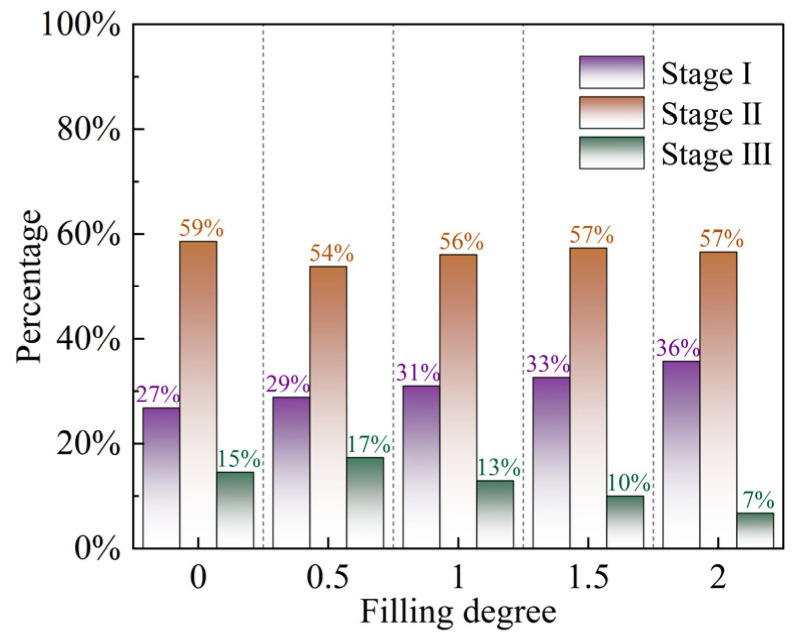
Characteristic figure of AE time distribution of bolted filled joint specimens.

The AE events of BFRP bolted filled joint specimens bolted with five filling degrees at J2 and $\sigma_{n}$ = 4 MPa were counted according to the occurrence time, and the results are shown in Figure 17, in which Stage I (0~200 s), Stage II (200~600 s), and Stage III (600~1000 s) correspond to pre-peak elasticity, post-peak plasticity, and residual strength stages, respectively, in Section 3.1 stage. As can be seen from the figure, the proportion of AE events in Stage I increases with the increase of Δ. This is because the AE events in this stage are generated by the closure of primary cracks and the expansion of microcracks within the rock, and the number of cracks sprouting in the filling layer is higher than that in the upper and lower blocks under the same conditions. It can also be seen from the figure that, regardless of the filling degree, the AE events generated by the damage of the bolted filled joint specimens were mainly concentrated in Stage II, which accounted for about the same proportion as the sum of the proportions in Stages I and III, which were 59%, 54%, 56%, 57%, and 57%, respectively. According to the previous analysis, when Δ is larger, the filling layer is heavily fragmented and the crush zone is larger, and this damage occurs mostly in Stage II, so the proportion of acoustic emission events in Stage II increases with increasing Δ. Some AE events are generated by the slip of the filled joints in Stage III, where the influence of the filling degree is not significant, so the percentage of AE in Stage III mainly depends on the first two stages.

Based on the above analysis, it can be seen that there is a good correspondence between the AE evolution characteristics and the shear failure process of the bolted filled joint specimen, which reveals the failure evolution process, the failure mechanism, and the change rule of the mechanical behavior of the rocks or joint surfaces in the shear experiment of the bolted filled joints. The AE energy value and the cumulative energy value of the AE quantify the degree of failure and failure of the specimen in the process of shear. The AE spatial distribution characteristics can intuitively reflect the spatial distribution and expansion path of cracks at each stage, and the AE time distribution characteristics verify the stage of specimen failure.

### 4.3. Discussion

This study confirms that BFRP bolts can effectively inhibit the shear slip of filled joints by virtue of their high tensile strength and controlled deformation characteristics (vertical deformation of 15.5–22.3 mm and horizontal deformation of 4.7–6.9 mm). Its progressive fiber fracture mechanism is superior to the brittle failure of traditional steel bolts, and it is especially suitable for the reinforcement of jointed rock bodies that require coordinated deformation. As underground construction continues to expand to the deep, the engineering challenges posed by the “three highs and one disturbance” (high geostress, high geothermal temperature, high karst water, and strong construction disturbance) are becoming more and more prominent, and future research should focus on optimization of high-stress working conditions and long-term performance.

## 5. Conclusions

In this paper, the effects of the degree of filling, joint roughness and normal stress on the shear mechanical properties and shear failure mode of intact specimens were investigated by carrying out the straight shear experiment of BFRP bolts bolted filled joint roughness and the shear failure properties of BFRP bolts were analyzed. Meanwhile, the AE energy evolution characteristics and AE distribution characteristics in the process of shear were systematically analyzed by combining them with the acoustic emission (AE) monitoring technology. The main conclusions are as follows:

1. Peak shear strength decreases with increasing filling degree, while normal stress, joint roughness and peak shear strength all show a positive correlation, which is due to the fact that they can directly or indirectly increase the engaging force of the filled joints.
2. The normal deformation of bolted filled joints is closely related to the filling degree, with the increase in filling degree, there are three kinds of evolution laws: first shear shrinkage and then shear dilatancy, first shear shrinkage and then shear dilatancy and then shear shrinkage, and shear shrinkage, and *JRC* and normal stress affect the degree of change in the specimen shear dilatancy-shear shrinkage.
3. The failure characteristics of the specimen are affected by the filling layer: the nodular bulge friction and gnawing failure when there is no filling, the filling layer friction and detachment with a small amount of nodular bulge gnawing failure when the filling degree is low, and the filling layer crumbles seriously when the filling degree is high.
4. BFRP bolts mainly play a shear capacity in the post-peak plastic stage, some fibers break in the process of shear, achieving the effect of “letting change”, and still have the ability to resist tensile and shear deformation in the later stage. With the increase in filling degree, the distance between A and B deformation critical points increases, and the deformation range of the bolt expands.
5. The acoustic emission energy evolution is closely related to the failure of the specimen. The pre-peak elasticity stage of the filled joint specimen basically does not cause damage, and the acoustic emission signal is relatively calm; the plasticity failure occurs mostly in the post-peak plasticity stage, where the acoustic emission energy surges and appears at its maximum, and then returns to be calm in the residual stage. The AE events are mainly concentrated in Stage II, the proportion of which is about the sum of the proportion of Stage I and Stage III, and this proportion increases with the increase in the filling degree.

## Figures and Tables

**Table 1:** Bolted filled joint shear experiment scheme.

Specimens	Normal Stress/MPa	Filling Degree	*JRC*
1~5	2	0.0, 0.5, 1.0, 1.5, 2.0	J2
6~10	4	0.0, 0.5, 1.0, 1.5, 2.0	J1
11~15	4	0.0, 0.5, 1.0, 1.5, 2.0	J2
16~20	4	0.0, 0.5, 1.0, 1.5, 2.0	J3
21~25	6	0.0, 0.5, 1.0, 1.5, 2.0	J2

**Table 2:** Peak shear strength at different normal stresses (J2).

	0	0.5	1	1.5	2
2 MPa	3.51	3.37	3.16	2.80	2.52
4 MPa	5.15	4.91	4.71	4.33	3.92
6 MPa	6.65	6.47	6.30	5.60	5.15

**Table 3:** Peak shear strength at different *JRC* (4 MPa).

	0	0.5	1	1.5	2
J1	4.85	4.48	4.24	4.11	3.80
J2	5.15	4.91	4.71	4.33	3.92
J3	5.97	5.01	4.97	4.80	4.24

## Data Availability

The original contributions presented in this study are included in the article. Further inquiries can be directed to the corresponding author.
